# Acute fatty liver of pregnancy: A case report of an uncommon disease

**DOI:** 10.4103/0972-5229.53115

**Published:** 2009

**Authors:** Kalpana S. Vora, Veena R. Shah, Geeta P. Parikh

**Affiliations:** **From:** Institute of Kidney Diseases and Research Center and Institute of Transplantation Sciences, Civil hospital campus, Ahmedabad-380 016, Gujarat, India

**Keywords:** Acute fatty liver of pregnancy, sepsis

## Abstract

A 24-year-old female at 34-week gestation, presented with malaise, nausea, vomiting, jaundice, and absent foetal movements. A clinical diagnosis of acute fatty liver of pregnancy was made. Although early caesarean section was performed, postoperative course was complicated by acute respiratory distress syndrome (ARDS) sepsis, and continuing coagulopathy. Supportive management in an intensive care unit resulted in successful outcome.

## Introduction

In pregnancy, pathological conditions causing abnormality of liver function tests need to be differentiated from normal physiologic changes. Among various causes of pathological hepatic dysfunction, acute fatty liver of pregnancy (AFLP) is uncommon compared to pre-eclampsia and hemolytic anemia, elevated liver enzymes and low platelets (HELLP) syndrome. Early diagnosis and prompt termination of pregnancy is necessary for better maternal and foetal outcomes.[1] We present a case report of a 24-year-old woman with AFLP complicated by sepsis and multiple organ dysfunction syndrome (MODS) requiring intensive care in spite of prompt termination of pregnancy.

## Case Report

A 24-year-old woman, G_1_P_1_L_0_ at 34-weeks gestation was admitted to the hospital with a history of absence of foetal movements for the past one day. She also complained of malaise, nausea, vomiting, and yellow colored urine since last ten days. Supportive treatment for acute viral hepatitis was given at a private clinic. She was detected to be hypertensive in the third trimester and was on tablet Alpha-methyl dopa 250 mg three times a day.

Physical examination revealed a well-nourished, somnolent, but easily arousable woman. Her temperature was 36.7°C, pulse rate was 104/min, respiratory rate 20/ min, and blood pressure 120/80 mmHg. She was oriented to person, place, and time, and her focal neurological findings were noncontributory. She was icteric, with mild edema of the legs. Abdomen was nontender. Ultrasonography revealed demise of the 34- week fetus and a fatty liver.

Complete blood counts revealed a hemoglobin: 11g/ dl, white blood cell count: 10,400/cumm, and platelet count: 57,000/cumm. Peripheral smear was negative for hemolysis and serum lactate dehydrogenase (S.LDH) levels were 238 mg%. Liver function tests showed aspartate aminotransferase: 208 U/l, alanine aminotransferase: 304 U/l, total bilirubin: 8.3 mg/dl, direct bilirubin: 6.7 mg/dl, alkaline phosphatase: 532 U/l, total protein: 6g/dl, and albumin: 2.6 g/dl. Biochemical tests revealed blood urea: 40 mg/dl, serum creatinine: 1.5 mg/dl, serum glucose: 60 mg/dl, and serum ammonia: 106 μmol/L. Coagulogram revealed a prothrombin time of 60 seconds with international normalized ratio (INR) of 3.2, fibrinogen: 62 mg/dl, and fibrin degradation products (FDP): 360 μg/ml. Urine analysis showed mild proteinuria. Serology tests like HBsAg, HCV, and HIV were all negative. A presumptive diagnosis of pre-eclampsia with HELLP and/or AFLP was made.

Considering the patient's increasing somnolence and absence of cervical dilatation, it was decided to perform an emergency lower segment caesarean section (LSCS) without a trial of labor. LSCS was performed three hours after admission under general anesthesia before which she received 10mg vitamin K, four units of platelet-rich plasma, and 10 units of cryoprecipitate to correct her coagulopathy. Intraoperatively, right internal jugular vein was cannulated and she received 1000ml crystalloids, six units of fresh frozen plasma (FFP), and two units of packed red cells. Her estimated blood loss was 600 ml. As her urinary output (u/o) was 50 ml and central venous pressure (CVP) remained between 8 and 10 mmHg, she was given 40 mg furosemide, after which her u/o increased to 100 ml. At the end of the operation, she was extubated after reversal when she followed commands. She was shifted to the ICU in view of the altered liver function, renal failure, and disseminated intravascular coagulation (DIC), and was put on broad-spectrum antibiotics. Twelve hours following admission to ICU, she became markedly tachycardic and tachypnoeic. Chest X-ray revealed pulmonary edema with CVP of 8mm Hg. An arterial blood gas sample on 4L/min of oxygen via a Hudson mask showed pH: 7.30, PCO_2_: 32mmHg, PO_2_: 102 mm Hg, bicarbonate: 15.0 mEq/L, and standard base deficit of 9.6 mEq/L. Since the patient was in acute renal failure with a urine output less than 25 ml/hour, she was dialyzed. For the next three days, she required intermittent dialysis for uraemia and replacement of blood components to correct coagulopathy. Although her renal failure improved with increasing u/o, she remained tachypneic and developed fever with a white blood cell count of 15000/cumm. Due to increasing respiratory distress and hypoxia, she was kept on controlled mechanical ventilation on day 4 of ICU admission. Chest X-ray at this point was suggestive of acute respiratory distress syndrome (ARDS) and she required high positive end-expiratory pressure (PEEP) to maintain PaO_2_ > 60 mmHg for 10 days. Her blood culture was positive for pseudomonas for which she received antibiotics as per culture and sensitivity report. Her bilirubin and liver enzymes started to decrease and platelets increased from day 10. During this period, she received 20 units of packed red blood cells (PRBC), 30 units of FFP, 40 units of platelets, and 20 units of cryoprecipitate to maintain an INR of <1.5, a platelet count of >50000/cumm, and fibrinogen >100 gm/dl. She made a gradual recovery and was weaned off from the ventilator on day 30 of admission to the hospital. Her liver and kidney function returned to normal and she was discharged on day 40.

## Discussion

Jaundice during pregnancy has many causes like cholestasis, cholelithiasis, viral hepatitis, pre-eclampsia with or without HELLP syndrome, and AFLP. Intrahepatic cholestasis of pregnancy may present during the third trimester but itching is the characteristic symptom and serum bilirubin concentration is rarely higher than 6mg/ dl. Cholelithiasis may occur at any time during pregnancy and is accompanied by pain in the right upper quadrant, and fever, and USG is usually diagnostic. Acute viral hepatitis in pregnancy presents as a systemic illness with fever, nausea, vomiting, fatigue, and jaundice, however, aminotransferase concentrations are markedly elevated (>500U/liter). All these causes were ruled out in our case on the basis of presentation, symptoms, and investigations.

Pre-eclampsia with liver involvement, HELLP syndrome, and AFLP manifest specific patterns, particularly in relationship with the timing of gestational age, however, they share many similarities in clinical features and laboratory abnormalities, and differentiation between them may be difficult. The manifestations of pre-eclampsia are usually observed in the second half of pregnancy, whereas the symptoms of HELLP syndrome and AFLP frequently appear in the third trimester.[1–3] The incidence of HELLP syndrome is much higher (1:5,000) than that of AFLP (1:13,000).[4] Severe coagulopathy, jaundice, hepatic encephalopathy, ascitis, hypoglycemia, and a mild to moderate elevation of transaminase levels are the key features of AFLP.[1–3,5–7] In our case, the clinical features of severe liver dysfunction appeared at the gestational age of 34 weeks. The symptoms initially mimicked those of acute viral hepatitis but clinical and laboratory evidence of severe coagulopathy, modest elevation of serum transaminase and bilirubin levels, hypoglycemia, an elevated ammonia value, and a low albumin level favored the diagnosis of AFLP over HELLP syndrome.

“Acute yellow atrophy of the liver,” a rare and fatal complication of pregnancy, was first described by Stander and Cadden in 1934.[8] The liver biopsy is diagnostic but is not always feasible especially in patients with severe coagulopathy[1] and it seldom influences acute management. Ultrasound and computed tomography have been used but the sensitivity and specificity of these imaging studies are insufficient to make a definitive diagnosis, and false negative results are common.[9] In our case, presence of coagulopathy did not allow us to perform liver biopsy. USG showed fatty changes and we did not perform CT scan as this facility was not available at our hospital and the patient was never stable enough to be transported to another center for investigation.

The definitive management of AFLP is rapid delivery of the fetus and supportive care. Usually jaundice, liver dysfunction, and DIC may progress for one to two days after delivery but will then improve.[9] Before 1980, both the maternal and fetal mortality rates were about 85%[10] and major causes were cerebral edema, gastrointestinal hemorrhage, renal failure, coagulopathy, and sepsis. Mortality has been reduced to less than 10% at present because of better recognition and appropriate management. Our patient presented rather late to us after the development of hepatic encephalopathy, acute renal failure, DIC, and fetal demise. Although we performed an early caesarean section, we were unable to interrupt the progression of the disease and her condition continued to deteriorate for 10 days with serious complications like ARDS, sepsis, and worsening coagulopathy. It is not clear whether ARDS occurred as a complication of acute liver failue (ALF), septicemia, or transfusion of multiple blood products, but it responded to supportive therapy.

In conclusion, AFLP is an uncommon, life-threatening complication of third trimester with variable presentation. While the natural history of the disease is improvement within 24–48 hours of delivery, it is recommended that patients who are critically ill at the time of presentation, who develop complications, or who continue to deteriorate despite emergency delivery, should be managed in the intensive care unit.

## References

[1] Bacq Y, Schiff ER, Sorrell MF, Schiff L, Maddrey WC (2006). The liver in pregnancy. Schiff's Diseases of the liver.

[2] Riely CA (1999). Liver disease in the pregnant patient. Am J Gastroenterol.

[3] Rahman TM, Wendon J (2002). Severe hepatic dysfunction in pregnancy. Q J Med.

[4] Pereira SP, O'Donohue J, Wendon J, William R (1997). Maternal and perinatal outcome in severe pregnancy – related liver disease. Hepatology.

[5] Reyes H (1999). Acute fatty liver of pregnancy: A cryptic disease threatening mother and child. Clin Liver Dis.

[6] Castro MA, Fassett MJ, Reynolds TB, Shaw KJ, Goodwin TM (1999). Reversible peripartum liver failure: A new perspective on the diagnosis, treatment, and cause of acute fatty liver of pregnancy, based on 28 consecutive cases. Am J Obstet Gynecol.

[7] Vigil-De Gracia PE (2001). Acute fatty liver and HELLP syndrome: Two distinct pregnancy disorders. Int J Gynecol Obstet.

[8] Stander H, Cadden B (1934). Acute yellow atrophy of the liver in pregnancy. Am J Obstet Gynecol.

[9] Usta IM, Barton JR, Amon EA, Gonzalez A, Sibai BM (1994). Acute fatty liver of pregnancy: An experience in the diagnosis and management of fourteen cases. Am J Obstet Gynecol.

[10] Kaplan MM (1985). Acute fatty liver of pregnancy. N Engl J Med.

